# A New Theory of Atmospheric Temperature

**Published:** 1860-03

**Authors:** Isaac Castleberry

**Affiliations:** Evansville, Indiana


### SELECTIONS.

### *A New Theory of Atmospheric Temperature.* By Isaac Castleberry, M. D., Evansville, Indiana.

Astronomy teaches that the sun is the centre of gravity and the source of heat. But the harmony and perpetuity of the physical world, seems to indicate that heat, as *heat*, does not emanate from the sun. The solar emanation is luminous and organizing; hence, I maintain the theory that, as the solar ray passes through ethereal space and impinges upon other forms of matter- it gives them properties which they did not possess, and receives, from its combination with these etherial forms of matter, elementary increments, out of which it elaborates two or three other cardinal forces as it approximates the earth.

This theory accords with the known law of heat, that its force diminishes in an inverse proportion to the squares of the distance, while the proximity of many comets to the sun cannot be reconciled to the theory that heat, as *heat*, is a solar emanation; for no substance, of which we have any knowledge, could have approached so near the sun as did the great comet, observed by Newton in 1682. No matter how great their tenuity, comets are material, and they have many of the attributes of materiality, such as density, gravity, and motive capacity. Did the caloric force, therefore, have augmented intensity commensurate with their proximity, which it must have according to the received laws of the radiation of heat, were it a direct solar emanation, it would consume or disperse the materials of which they are composed.

Again: astronomers assure us that comets are neither repelled nor driven to pieces by the caloric force, but that, on the contrary, several are known to approach the sun. It is true some perturbate, but this perturbation may be accounted for upon the principle that they are passing through a resisting medium.— Can these facts be reconciled to the received theory that the caloric force emanates from the sun? Heat has been demonstrated to be everywhere governed by the same uniform law: why, then, are not some of the nearer planets to the sun, over-heated? As the laws of the universe are simple and uniform, may it not be that the solar ray is at first only luminous, and that it acquires the caloric and chemical forces as it approaches the earth?—Chemistry clearly reveals that the solar ray is divisible into three forms of force, luminous, caloric, and chemical; and that the cardinal forms of force which pervade the atmosphere, are light, heat and electricity. These are intimately correlated, and mutually convertible into the fragmentary forms of force by which the functions of animals and vegetables, and the changes of inorganic matter, are created, maintained, and governed.

The atmosphere is an immense ocean of oxygen and nitrogen, with a trace of carbonic acid; and, according to this theory, the instant the luminous force of a solar ray impinges upon it, a series of chemical actions begin to undulate through it, which augment in intensity as they approach the earth; increments of heat, chemical force, and electricity are developed, and the conditions necessary to sustain vegetable and animal life are created. The physical sciences most clearly indicate that electricity, and the caloric, and the chemical forces are universally present throughout the atmosphere, that they pervade animate and inanimate matter, and that the organic functions of the vegetable and animal kingdoms and the physical manifestations of inorganic matter are dependent upon the quantitities and states of these forces for their power. In what mode soever these forces endow organized bodies with the functions by which they are enabled to form and renew their compositions, they must determine the actions which modify their structures and functions, for the elements of which these bodies are composed, are the same in all parts of the world. The elements of water, the air, and the surface of the earth, are the same in South America as at Melville Island, the same in Europe as in America. The number of their species, the magnitude of their forms, and the complexity of their organizations, must, therefore, be regulated by the power of the forces which cause their development.

The physical manifestations of the electric, the caloric, and chemical forces diminish from the equator to the poles; so does the magnitude of animals and vegetables. Is it not evident, from these facts, that the power of living bodies to maintain and renew their compositions by assimilation is governed by these forces? The torrid zone has its mighty elephant and its huge hippopotamus, its luxuriant vegetation, and its exuberant foliage; the temperate zones have their noble horses and faithful oxen, their giant oaks and their lofty pines; and the frigid zones have their fleet reindeer and their crafty foxes, their stinted birch, and their dreary wastes of snow and struggling vegetation. In the torrid zone, man is effeminate and debased, ignorant and treacherous; in the temperate, athletic and noble, intelligent and patriotic; in the frigid, low and crafty, rude and unfaithful.

Thus physical causes in every latitude make their distinguishing impress on man, animals, and vegetation; yet the composition of the atmosphere they breathe and the water they drink, are composed of exactly the same elements. The physical sciences should, therefore, be earnestly cultivated, for they will reveal many of the mysteries both of health and disease. But I apprehend some of the fundamental laws of these sciences are at present based upon error.

The received theory of the distribution of heat is certainly erroneous, for it is based upon the hypothesis that, as the atmosphere is rare and diaphanous, but a small quantity of the heat of the solar rays which traverse it is retained; and that, as the denser and more inferior strata heated by the surface of the earth expand, they ascend and grow colder by the heat passing into a *latent* state. The *latency* of heat is an absurdity, which is perpetuated by the want of a knowledge of its convertibility into other forces. We know when winds prevail, the elements of the atmosphere are driven about by mechanical force; that this force is derived from heat; and that the motive intensity of the winds is in proportion to the quantity of heat lost. This is fully illustrated by the diminution of temperature which follows the gentle breeze, the chilling blast, or the sweeping tornado. Hence I affirm heat never exists, as *heat*, in a *latent* state, but that, when not sensible, it is transmuted into some other force and manifested as such. From what other source, except heat, could the winds be obtained? The force by which it is formed emanates from the sun, and is governed by uniform laws. How does the climate of the United States affect these laws?

This inquiry has never been answered philosophically, although it has engaged able minds, and learned theories have been advanced. But the conversion of heat into other forces was not known by them, nor were the experiments of Bache, upon heat, read by them, or they would have had a more comprehensive knowledge of this force. It is a well established fact that in this country the summer heat is everywhere intense, and from fifteen to twenty degrees higher than that in Europe. Why is this?—On the Atlantic coast there is an unascertained prolongation of the continent towards the north pole, and an ocean current sweeping immense masses of ice southward. A continuous current of cool air from this polar stream has been assigned as the cause of the low temperature of the New England States, a temperature much lower than that in the same latitudes in Europe. The Gulf stream is supposed to have a potential influence in elevating the temperature of Europe. It stretches across the Atlantic between Cape Hatteras and the Azores, forming nearly in the middle of the Atlantic, a lake of warm water, which is estimated by some authors to be equal in extent to the Mediterranean. The warm air of this ocean lake is wafted over the coast of western Europe. (*Fourey*, *Climate U. S.*, p. 91).

A principal cause of the high temperature of the Pacific coast, is attributed to the steady prevalence of western winds, because there is thus swept from the ocean, which never sinks below the freezing point, a humid atmosphere. This, in its passage over the land, has a constant tendency to establish an equilibrium of temperature, and as its vapor is gradually condensed it evolves heat. These physical conditions of the Atlantic and Pacific coasts modify the meterological phenomena of the New England States and of Oregon and California. But what modifies the climate of the Mississippi valley? Does physical geography answer this enquiry?

The Cordillerian chain of mountains on the west, impedes or arrests the warm, moist wind from the Pacific, while the Apilachian chain does not shelter it from the cold north-east winds. The south-west wind lessens the fiery heat of the summer and early autumn; but the north-east wind hurries, in freezing blasts, from his icy home in the arctic regions, to bring over the Missisippi valley hoar frosts and dreary desolation. Local causes within the valley, also, produce a potential influence upon the climate; for the western table-lands, rising gradually to the height of 600 or 800 feet, cause no manifest diminution of temperature; yet, according to Humboldt, "elevations of 400 metres [1312 feet] appear to have a very sensible influence on the mean temperature, even when great portions of countries rise progressively." Gernier, Laplace, Gay Lussac, Prout, of Europe, and Dr. Drake, of this country, concur in the opinion that 300 or 400 feet of elevation will cause Fahrenheit's thermometer to sink one degree. According to these observers, therefore, the elevated land in our western States, should produce a diminution of temperature equal to two or three degrees. But we have seen that an elevation of 600 or 800 feet on our Western table-lands has little or no influence. Other causes must, therefore, be assigned.

The Mississippi, with its hundred tributaries, affords an ample highway for the commerce of an opulent people, and promotes the fertility of the most productive valley on the globe. But the floods of these rivers often transcend the limits of utility, and entail upon the adjacent country the effects of inundation which frequently modify the geological formations of the soil and its physical condition. In the "bottoms," which are rich in decomposing organic remains, vegetation attains exuberant growth and foliage of prodigious size. When these are mature, they are deposited; and when the necessary conditions exist for their rapid decomposition, an immense series of chemical affinities are developed. These will be commensurate with the degree of heat and moisture. The alluvial lands of the Mississippi and its tributaties abound in sluggish bayous and ponds of stagnant water.—When these are inundated by a spring freshet, they constitute so many laboratories for the decomposition of organic remains, and for the generation of atmospheric vicissitudes. As they receive the summer heat, a wonderful manifestation of chemical affinities transpires. These augment in intensity as the degree of heat increases, and the moisture of the alluvial soil and the quantity of stagnant water diminish by evaporation. What are some of the products of these paludal laboratories? Some of the caloric forces of the solar rays are transmuted into electricity, by which the elements of the animal and vegetable compounds are liberated from former combination. Water facilitates this decomposition. When it is devoid of organic constituents, the caloric and chemical forces have, however, only a feeble affinity for it, and decomposes it slowly. But water upon the surface of the earth, in sluggish streams, and in alluvial ponds and bayous, always abounds in decomposing compounds of organic remains, for which the caloric and chemical forces have a strong affinity.—Hence, these forces freely pervade water made impure by vegetable and animal compounds in a state of decomposition, and its temperature becomes proportionately elevated.

The moment they impinge upon water in this condition, a part of the caloric force is transmuted into mechanical force, by which some of the aqueous atoms are driven apart, and others into the atmosphere in the form of vapor, and another part of the caloric force is transmuted into electricity, by which the organized compounds are decomposed; while the chemical force is divided into an immense series of fragmentary forms of chemical action between the decomposing and inchoate compounds. The circumambiant atmosphere gradually becomes more or less loaded with moisture, in which the liberated gases are commingled, and as the solar rays struggle through, they augment the quantity of electricity and impart increased intensity to the chemical actions.

Where the gases ascend to freedom, increments of each are in a nascent state, in which state they always seek with avidity for new associates. Some of these are readily found entangled in the moisture of the atmosphere, while others must be sought for within the exuberant foliage of plants and trees. As the solar rays impinge upon the aqueous and gaseous masses floating about tumultuously, the chemical force gives increased energy to the nascent oxygen, which now endeavors to attain its former supremacy over the gaseous elements; but some of these are so allied to the moisture in the atmospere that it can not obtain access to them with sufficient facility to prevent their combination with each other. This union of nascent gases without the primary influence of oxygen, may result in the formation of neutral compounds, as ammonia; or in deleterious gases, as sulphuretted hydrogen, carburetted nitrogen, etc. When free gases are not consumed by combination with each other, or by the foliage of the plants and trees, they are extremely prejudicial to the growth and maturity of fruits and the health of mankind. The *fevers* of man, the *rust* upon vegetables, and the *speck* upon growing and maturing fruit, bear ample evidence of this. Sulphuretted hydrogen, carburetted nitrogen, carbonic acid, and moisture in the atmosphere, have each been regarded as *the* cause of fever by some physicians, while oxygen and electricity have either escaped observation, or been esteemed almost innocent. But many other physicians have overlooked this condition of the atmosphere and gazed fixedly upon a myth called *malaria*, with whose *fancied* attributes they have been entranced for life.

The undulating lands of the north and south-western States, which are neither enriched nor despoiled by river freshets, affording fields for fruitful cultivation and prairies of luxuriant vegetation, variegated by dense forests attired in gorgeous foliage, are not exempt from the disturbing or deleterious effects of an over-supply of positive electricity and free gases, developed by the chemical and caloric forces undulating through a moist atmosphere and impinging upon a humid soil abounding in decomposing vegetable and animal remains. But, for obvious reasons, the excess of electricity and gases is not so great as in alluvial lands, and therefore not so prejudicial to the health of mankind nor so injurious to fruits, because the water upon the soil is generally evaporated before the maturity of the plants and foliage, by the decomposition of which the electricity and gases are chiefly generated. It is true, vegetation may die prematurely, and some of that which matured the previous year may not have been fully decomposed, and then a condition will exist, by the addition of moisture favorable to the disengagement of gases and to the development of electricity. But, unless unusual conditions exist, the quantities developed will not be much more than is consumed by the surrounding plants and foliage, whose gorgeous hues and ample size give evidence of a generous supply of appropriate nourishment.

When copious rains fall in June and July, and August and September are unusually dry, or when all these months are alternately very wet and extremely dry, immense quantities of electricity and gases will be developed, because the maturity of early vegetation favors its decomposition; and as the quantity of water upon the soil becomes more and more contaminated by decomposing organic remains, its evaporation will be increased proportionately; then the atmosphere becomes loaded with warm moisture, which is largely intermixed with nascent and free gases, vegetation *rusts*, fruit *specks*, and man *sickens*.

The unascertained prolongation of the Atlantic coast towards the north pole, along which an ocean current sweeps southward, carrying huge icebergs, and attended by chilling winds; the lofty range of the Rocky Mountains, which shelter the Pacific coast from the polar winds, and the constant prevalence of warm, moist western winds upon that coast, most unquestionably modify the climates of these coasts.

But these physical conditions do not solve the philosophical problem that the temperature of this country is several degrees higher in summer and several degrees lower in winter than that of corresponding latitudes in Europe. As physical geography does not afford us any adequate explanation of this discrepancy between the temperature of Europe and America, a solution must be sought for in local conditions. It is a well established principle in chemistry that the combination of oxygen and carbon always evolves heat, and that the quantity of heat evolved is always equal to the quantity of oxygen and carbon consumed. The amplification of this principle fully illustrates the increased temperature of this country; for, as the spring sun falls upon the earth, the congealed coffers of the organic remains of former years are unbosomed, oxygen slowly combines with carbon, and millions of fires are kindled. The advancing sun increases the quantity of oxygen and carbon liberated, which manifest a proportionately increased affinity for each other; the fires grow warmer and warmer. Heat begins to radiate from them; a series of chemical actions transpire in the vegetable kingdom; sap rises and buds expand; the earth is soon robed in verdure.

At the summer solstice the sun is nearest the earth; the luminous force descends brilliantly—vapors are dispersed rapidly—a serene and gleaming sky oppresses the beholder; the early leaves and flowers are matured, the fervid heat of the atmosphere hastens their decomposition; its oxygen passes readily into the fibrous structures of the fallen vegetable tissues, and combines freely with their carbon; a proportionate combustion ensues, and an equivalence of heat is evolved.

Late in July and early in August storms of rain usually occur. These accelerate the maturity of plants, leaves and fruits, and for a few days diminish the temperature of the atmosphere. But the rains cease—the vapors disappear—the sky is serene—the sun glows intensely—the vegetable kingdom presents a sublime conflagration. The matured fruit and fallen plant of the fruitful plain and teeming valley supply the fuel abundantly. These are the hottest days of the year. Why? Not, certainly, because the sun is nearest to the earth—for he has passed the solstice.—Chemistry answers the inquiry philosophically, and the physical manifestations of heat throughout the universe, confirm her thermal statements. And were the observations carefully made, the quantity of heat manifested in any latitude would be found to bear a close and dependent relation to the quantity of oxygen and carbon consumed.

The luxuriant vegetation, the exuberant foliage, and the dense forests of the Mississippi valley, in a state of almost unceasing combustion, must generate an immense quantity of heat; for I have already stated that it is a well established principle in chemistry that the combination of oxygen and carbon always produces an amount of heat equal to the quantities of these elements consumed. Did the combustion take place more rapidly, or in a more limited space, the heat generated would be sensibly manifested; but the quantity produced is not the less because the combustion takes place slowly over an immense expanse of territory. Should not the thermometer exhibit this increased quantity of heat? That is precisely what it does on the western table-lands, and over the American continent generally. It also affords a physical exposition of the observation that in this country gradual elevation to the extent of six hundred or eight hundred feet, does not produce the same diminution of temperature as in Europe.—*Cincinnati Lancet and Observer.*

